# Correlating drug prescriptions with prognosis in severe COVID-19: first step towards resource management

**DOI:** 10.1186/s12911-022-01983-7

**Published:** 2022-09-21

**Authors:** Anna S. Levin, Maristela P. Freire, Maura Salaroli de Oliveira, Ana Catharina S. Nastri, Leila S. Harima, Lauro Vieira Perdigão-Neto, Marcello M. Magri, Gabriel Fialkovitz, Pedro H. M. F. Figueiredo, Rinaldo Focaccia Siciliano, Ester C. Sabino, Danilo P. N. Carlotti, Davi Silva Rodrigues, Fátima L. S. Nunes, João Eduardo Ferreira, HCFMUSP COVID-19 Study Group, Geraldo Busatto Filho, Eloisa Bonfá, Edivaldo Utiyama, Aluisio Segurado, Beatriz Perondi, Anna M. Morais, Amanda Montal, Solange Fusco, Marjorie Fregonesi, Marcelo Rocha, Izabel Marcilio, Izabel C. Rios, Fabiane, Y. O. Kawano, M. Amelia de Jesus, Esper G. Kallas, Carolina Marmo, Clarice Tanaka, Heraldo P. de Souza, Julio F. M. Marchini, Carlos Carvalho, Juliana C. Ferreira, Thais Guimaraes, Carolina S. Lazari, Alberto J. S. Duarte, M. Cristina Braido, P. B. Francisco, Silvia F. Costa

**Affiliations:** 1 Department of Infectious Diseases, Faculdade de Medicina, Universidade de São Paulo, São Paulo, Brazil; 2 Department of Infection Control, Hospital das Clínicas, Universidade de São Paulo, São Paulo, Brazil; 3 Division of Infectious Diseases, Faculdade de Medicina, Universidade de São Paulo, São Paulo, Brazil; 4 Clinical Director’s Office, Hospital das Clínicas, Faculdade de Medicina, Universidade de São Paulo, São Paulo, Brazil; 5 Núcleo de Vigilância Epidemiológica, Hospital das Clínicas, Faculdade de Medicina, Universidade de São Paulo, São Paulo, Brazil; 6 Computer Science Department, Institute of Mathematics and Statistics, Universidade de São Paulo, São Paulo, Brazil; 7 Laboratory of Computer Applications for Health Care; School of Arts, Sciences and Humanities, Universidade de São Paulo, São Paulo, Brazil; 8 Hospital das Clínicas da Faculdade de Medicina, Universidade de São Paulo, São Paulo, Brazil

**Keywords:** Severe COVID-19, Treatment, Hospitalization, Prognosis

## Abstract

**Background:**

Optimal COVID-19 management is still undefined. In this complicated scenario, the construction of a computational model capable of extracting information from electronic medical records, correlating signs, symptoms and medical prescriptions, could improve patient management/prognosis.

**Methods:**

The aim of this study is to investigate the correlation between drug prescriptions and outcome in patients with COVID-19. We extracted data from 3674 medical records of hospitalized patients: drug prescriptions, outcome, and demographics. The outcome evaluated was hospital outcome. We applied correlation analysis using a Logistic Regression algorithm for machine learning with Lasso and Matthews correlation coefficient.

**Results:**

We found correlations between drugs and patient outcomes (death/discharged alive). Anticoagulants, used very frequently during all phases of the disease, were associated with good prognosis only after the first week of symptoms. Antibiotics very frequently prescribed, especially early, were not correlated with outcome, suggesting that bacterial infections may not be important in determining prognosis. There were no differences between age groups.

**Conclusions:**

In conclusion, we achieved an important result in the area of Artificial Intelligence, as we were able to establish a correlation between concrete variables in a real and extremely complex environment of clinical data from COVID-19. Our results are an initial and promising contribution in decision-making and real-time environments to support resource management and forecasting prognosis of patients with COVID-19.

**Supplementary Information:**

The online version contains supplementary material available at 10.1186/s12911-022-01983-7.

## Background

The disease caused by the novel coronavirus (SARS-CoV-2) was first recorded in December 2019, in Wuhan, Hubei province, China. The COVID-19 pandemic was recognized by the World Health Organization (WHO) on March 11, 2020 and the first case in Brazil was reported on February 26, 2020. Up to May 2022, almost 515 million cases of COVID-19 and over 6 million deaths due to the disease had been reported [1]. The death rate among patients who require hospitalization varies between 11 and 15% [2]. This scenario reveals the extreme severity of the disease, leading to a crisis in the global health system and a very negative socio-economic impact. The impact of COVID-19 in Brazil has been disastrous, leading to almost 22 million cases and more than 600,000 reported deaths [1].

Although many advances in the knowledge of the disease have been reported daily, COVID-19 continues without proven specific treatments. The disease is associated with inflammation and a prothrombotic state, and the use of systemic corticosteroids in patients in need of oxygen therapy is now considered standard in clinical management. Anticoagulants and antiplatelet therapy have shown beneficial effects based on the severity of the disease [3, 4].

In this complicated scenario for the management of hospital resources, the construction of a computational model capable of extracting information from electronic medical records, in order to correlate signs, symptoms and medical prescriptions more easily, could improve resource management and prognosis of these patients and contribute to the solution of this major public health problem.

Models have been used to observe how variables, such as drug prescriptions, are correlated with the outcome [5–8]. However, finding relationships between drug prescriptions and patient outcomes is still an underexplored issue. The advance of technology in the last years has allowed Artificial Intelligence (AI) to emerge as a useful, viable, and efficient approach to discovery knowledge in huge amounts of data. By using techniques to standardize the data and methods to discover relations between variables, it is possible to discover which type of drug prescriptions are correlated to a patient’s prognosis.

The aim of this study is to investigate the correlation between drug prescriptions and outcome in patients with COVID-19.

## Materials and methods

First we extracted data from electronic medical records, especially drug prescriptions, outcome and demographic information. After that, the drug prescriptions were grouped using medical knowledge. Finally, we applied correlation analysis using Logistic Regression with Lasso and Matthews correlation coefficient. The results were summarized using data visualization techniques.

### Setting

Hospital das Clínicas (HC) is a public teaching hospital located in São Paulo. It comprises seven buildings with 2200 beds and 22,000 employees. The hospital was designated by the Sao Paulo State government to receive the severe cases of COVID-19. The Central Institute (CI) is an 11-floor building with 6000 healthcare workers designated to receive all the COVID-19 cases referred to the hospital. It included an emergency unit, 300 ICU beds, and 300 beds in regular wards, and was entirely dedicated to COVID-19 care [2].

This is an observational cohort study evaluating COVID-19 patients who were admitted to the Central Institute between March 30 and August 31, 2020.

### Participants

Inclusion criteria: patients hospitalized in CI from March through August, 2020 with COVID-19 according to the following definition:

- At least one of the following symptoms: cough, fever, shortness of breath, sudden onset of anosmia, ageusia or dysgeusia
- AND one of the following:
  - Radiological evidence showing lesions compatible with COVID-19 (e.g. bilateral, peripheral ground-glass opacities).
  - Positive RT-PCR or antigen test for SARS-CoV-2 in a clinical respiratory specimen.

### Database

Drug prescriptions were extracted from patients’ electronic health records (EHR) on a daily basis for the entire length of their hospital stay. Medical records also contained daily medical and nurse evaluations, demographics, vital signs, laboratory tests, written evaluations of radiologic films, and prescriptions.

For each patient, prescriptions were divided into 4 time periods, starting from the date of the onset of symptoms: first week, second week, third week, and $\ge$ fourth week. The patients for which the date of onset of symptoms was not available were excluded.

Data was also stratified by age group: $\le$ 50 years; 51 to 60 years; from 61 to 70 years; and > 70 years.

Drug prescriptions were grouped into the following categories: non-steroidal anti-inflammatory, antibiotics, anticoagulants, anticonvulsants, antifungals, antivirals, anti-hypertensive alpha blockers, anti-hypertensive alpha and beta-blockers, anti-hypertensive beta-blockers, anti-hypertensive calcium channel blockers, angiotensin receptor blockers (ARB), angiotensin-converting enzyme inhibitors (ACEi), anti-hypertensive vasodilators, antipyretic, proton pump inhibitor, neuromuscular blocking agents, intravenous corticosteroids, oral corticosteroids, vasoactive drugs, statins, blood components, immunosuppressive drugs, sedatives, anti-psychotics, electrolytes and vitamins. Other drugs that did not belong to any of these categories were evaluated separately.

The observed outcome was in-hospital mortality.

### Data extraction

We obtained the following data about the patients: the date of onset of their symptoms, their age, the dates of when each drug was administered, the date and condition in which the patient left the hospital (dead or discharged alive).

In order to test the correlation between the administration of each drug, or class of drug, and the observed outcome a series of four experiments was devised, corresponding to the intervals between the onset of symptoms and the administration of the drugs.

For each patient drugs were evaluated based on the time they were administered: in the first, second, third, or fourth week of the onset of symptoms. The absolute frequency of people to whom the drug was administered and their outcome were registered.

### Correlation between drug prescription and outcome

To investigate the correlation between the drug prescriptions and the outcome, we used two complementary regression methods: Logistic Regression with Lasso and Matthews correlation coefficient. The former aims to identify the relative importance of each drug and their correlation with the outcome [9, 10].

The first algorithm was used to predict the outcome of a binary classification problem given a set of independent variables. When a specific limitation is applied, known as Lasso, it forces the algorithm to limit the absolute importance of the beta of the variables, reducing the number of variables considered by the model and thus selecting the most important features. The beta is the name used to denote the coefficient of the variables after the regression converges. The bigger the coefficient, the more relevant the variable is to the correlation. When this algorithm is applied to a dataset that was previously classified the result can be interpreted as means of selecting the most important features and identifying their joint correlation with the outcome. The most important difference of this method and the second method used is that this method considers all the variables and the outcome together, this being the main reason for its use. The objective is to test which variables, when considered together, are most likely correlated with the result. There is no standard scale for the scores or result. The higher the scores, the more correlated is the administration of the drug and the death of the patient. The smaller the number, greater is the correlation with the patient being successfully discharged.

The second correlation algorithm used (Matthews correlation coefficient) has the same definition as the Pearson correlation coefficient [11]. This algorithm was created to test for the correlation of two binary variables. There are only two possible outcomes and this coefficient expresses in the interval [$-1, 1$] if the two vectors are correlated. A zero value indicates that there is no correlation between them. The interval extremes indicate perfect positive correlation or perfect inverse correlation (when the value is negative). In the case of the present study, one vector represents a particular drug that was administered or not administered to a given patient and the outcome is whether this patient died or was discharged. The bigger the number, greater is the correlation between the administration of the drug and the death of the patient. The smaller the number, the greater the correlation with the patient being successfully discharged.

This second algorithm was used to test the individual correlation of variables with the outcome. This was done to allow a more traditional approach of observing how the patients react after receiving or not receiving a certain drug. Since there are interactions between drugs and since there are drugs only administered to patients who are well or really ill, there is a natural correlation between drugs and patients’ health. This can only be observed and considered by the medical community, since we do not have, at this moment, any information about the patient’s symptoms.

The implementations of the algorithms used can be found in scikit-learn library [12].

We used different information visualization techniques [13] to present the results found. A broad view is provided with bubble charts and a more detailed perspective is presented via tables. We selected, processed and ordered relevant data to build charts and tables using Plotly [14] and matplotlib [15] modules for Python.

## Results

Between March and August 2020, 3776 patients with confirmed COVID-19 were admitted to the hospital. Of these, 102 were excluded from the study due to the lack of clinical and epidemiological data. Thus data of 3674 were analyzed.

**Table 1:** Demographic characteristics of 3674 patients admitted to the hospital with confirmed COVID-19, and drugs administered to them during their entire hospitalization (Hospital das Clínicas, University of Sao Paulo, Brazil—30 March–30 August, 2020)

Demographic data	N: 3674
Female sex, n (%)	1682 (46%)
Age (years)	
Mean (SD)	58 (17.9)
Median (range)	60 (13–101)
Days of hospital stay	
Mean (SD)	14.6 (1.3)
Median (range)	10.3 (1–153)
Admission directly to an ICU, n (%)	1475 (40%)
Deaths	1169 (32%)

Number of prescriptions evaluated	821,532
Drugs	N (%)
Anticoagulants	3445 (94)
Antibiotics	3300 (90)
Antipyretics	3010 (82)
Proton pump inhibitor	2556 (70)
Sedatives	2002 (54)
Electrolytes	1958 (53)
Furosemide	1916 (52)
Insulin	1700 (46)
IV corticosteroids	1652 (45)
Vasoactive drugs	1572 (43)
Antipsychotic	1458 (40)
Bisacodyl	1360 (37)
Neuromuscular blocking agents	1278 (35)
Oral corticosteroids	1215 (33)
Antiviral drugs	1160 (32)
Ondansetron	849 (23)
Metoclopramide	819 (22)
Anticonvulsive drugs	751(20)
Hydrochlorothiazide	739 (20)
Statins	728 (20)
Anti-hypertensive calcium channel blocker	660 (18)
Acetylsalicylic acid	660 (18)
Methadone	629 (17)
Blood components	622 (17)
Angiotensin-converting enzyme inhibitors	519 (14)
Angiotensin receptor blockers	508 (14)
Vitamins	505 (14)
Atropine	503 (14)
Salbutamol	488 (13)
Scopolamine	447 (12)
Amiodarone	431 (12)
Dimethicone	431 (12)
Anti-hypertensive beta-blocker	399 (11)
Levothyroxine	392 (11)
Anti-hypertensive vasodilator	352 (10)
Ivermectin	312 (8)
Hydroxychloroquine	43 (1)
Tocilizumab	3 (0.1)

*SD* standard deviation, *ICU* intensive care unit, *IV* intravenous

Demographic characteristics of the patients are shown in Table 1. The in-hospital mortality rates were: 49% (724/1475) among patients who were admitted directly to an ICU; and 20% (441/2199) among patients who were admitted to a ward P < 0.001 (HR 1.43; 95% CI 1.26–1.61). 821,532 prescriptions of administered drugs were included in the analysis.

**Fig. 1:**
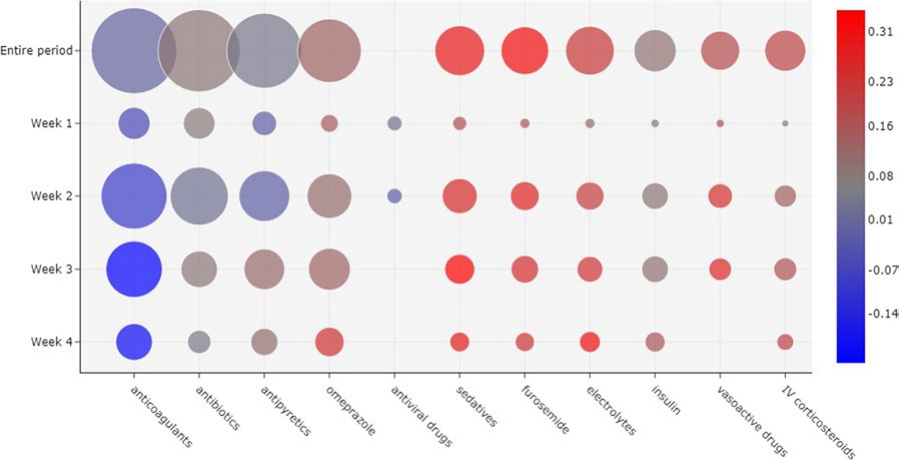
Correlation between the 10 most prescribed drugs and outcome, considering all the patients evaluated. The size of a bubble indicates how frequently a drug was administered. The color of a bubble indicates the Matthews correlation of the drug with death (shades of red) or discharge (shades of blue). A color scale is provided to indicate the numerical correlation (from $-1$ to $+1$). The weeks are considered based on the onset of symptoms. (*IV* intravenous)

Patients’ prescriptions were evaluated for each week considering the date of the onset of symptoms. Prescriptions of 1476 (40.2%) patients were evaluated in the first week of symptoms; 2871 (78.1%) on the second week; 2467 (67.1%) on the third week; and 1598 (43.5%) patients were evaluated on the fourth week and later. Figure 1 shows a bubble chart per week since the onset of symptoms with the ten most prescribed drugs. There is, for example, a strong correlation between the administration of furosemide and patient death, especially in weeks 3 and 4. On the other hand, there is a strong correlation between the administration of anticoagulants and patient discharge, especially in week 3 and later. Antivirals were the fifth most prescribed drugs during the first week, but were not frequently administered in later weeks or considering the entire period. In contrast, vasoactive drugs were one of the ten most prescribed drugs in general and in the first three weeks, but not in week 4 and later.

**Fig. 2:**
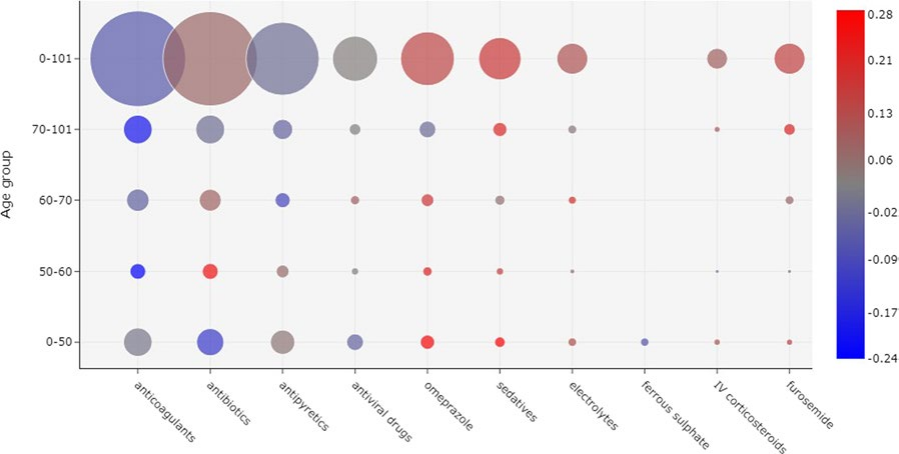
Correlation between drug administration and outcome, considering the 10 most frequently prescribed drugs in week 1 of the onset of symptoms. The prescriptions were divided according to patient age groups. The size of a bubble indicates how frequently a drug was administered. The color of a bubble indicates the Matthews correlation of the drug with death (shades of red) or discharge (shades of blue). A color scale is provided to indicate the numerical correlation (from $-1$ to $+1$). (*IV* intravenous)

A visualization of the most prescribed drugs according to age group in week 1 is shown in Fig. 2. In general, anticoagulants were the most frequently administered drugs in the first week and were more correlated with patient discharge, but there were differences between age groups. For patients between ages 50 and 60, anticoagulants were more correlated to patient discharge. A similar correlation was found for patients between ages 70 and 101, although with a greater frequency of administration. For the remaining age groups, and in general, no correlation between anticoagulants and outcome was observed.

Antibiotics were the second most frequently administered drugs in week 1. For patients between ages 50 and 60, these drugs were strongly correlated with death, but were less frequently administered in comparison to the other age groups. Yet, for ages between 0 and 50, antibiotics were more correlated with patient discharge and more frequently prescribed.

**Fig. 3:**
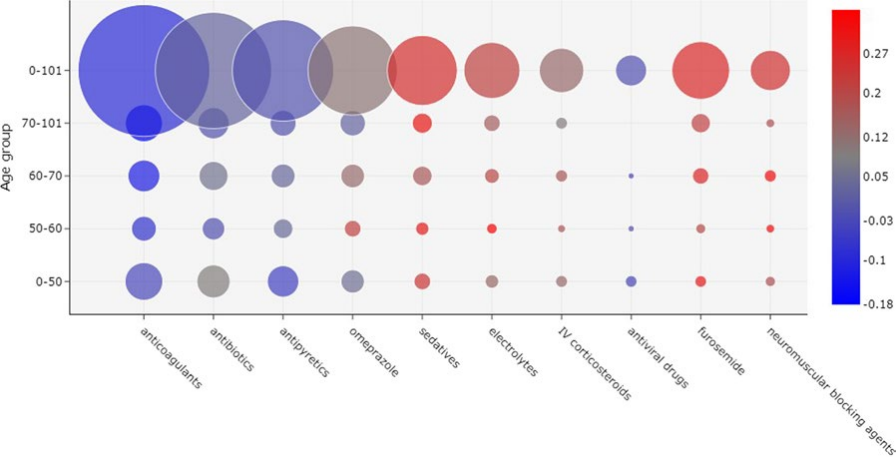
Correlation between drug administration and outcome, considering the 10 most frequently prescribed drugs in Week 2 of the onset of symptoms. The size of a bubble indicates how frequently a drug was administered. The color of a bubble indicates the Matthews correlation of the drug with death (shades of red) or discharge (shades of blue). A color scale is provided to indicate the numerical correlation (from $-1$ to $+1$). (*IV* intravenous)

Figure 3 shows a bubble chart with the most frequently administered drugs in week 2. Similarly to week 1, anticoagulants were the most prescribed drugs for all age groups. However, in contrast to the first week, these drugs were correlated with patient discharge in all age groups, especially for ages 60 and above.

**Fig. 4:**
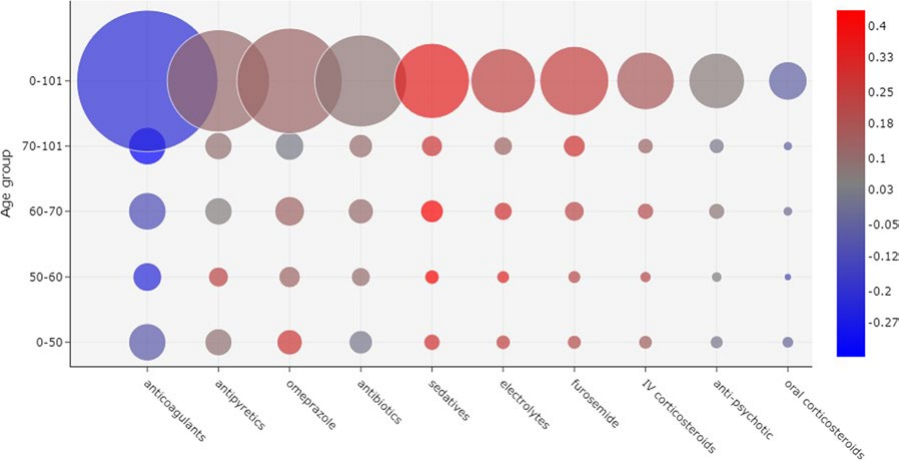
Correlation between drug administration and outcome, considering the 10 most frequently prescribed drugs in week 3 of the onset of symptoms. The size of a bubble indicates how frequently a drug was administered. The color of a bubble indicates the Matthews correlation of the drug with death (shades of red) or discharge (shades of blue). A color scale is provided to indicate the numerical correlation (from $-1$ to $+1$). (*IV* intravenous)

**Fig. 5:**
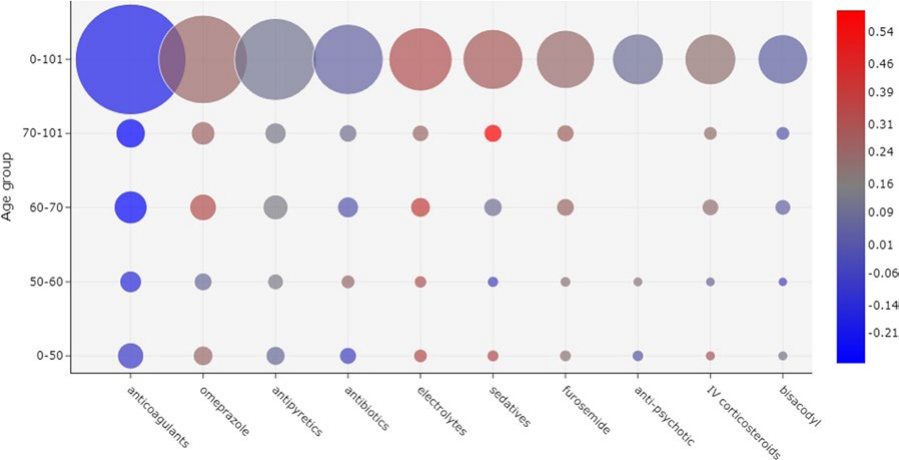
Correlation between drug administration and outcome, considering the 10 most frequently prescribed drugs in week 4 of the onset of symptoms. The size of a bubble indicates how frequently a drug was administered. The color of a bubble indicates the Matthews correlation of the drug with death (shades of red) or discharge (shades of blue). A color scale is provided to indicate the numerical correlation (from $-1$ to $+1$). (*IV* intravenous)

Figures 4 and 5 show the most frequently administered drugs in weeks 3 and 4, respectively. As before, anticoagulants were the most prescribed drugs and were correlated with patient discharge.

Feature selection is a process, as described, which selects the features that are most correlated with the outcome. The way the coefficients are calculated by the algorithm Logistic Regression with Lasso makes it so that a week or an age group is not comparable with others, differently from the Matthew’s correlation previously presented. However, this is an algorithm capable of classifying patients given the set of drugs administered and therefore may provide insights. It can be combined with other algorithms to improve its predictive power and further help in the decision making process of medical practitioners.

**Table 2:** Correlation analysis between drug administration and patient outcome with feature selection using Logistic Regression with Lasso algorithm, and considering all weeks and all age groups. The table displays the 10 prescribed drugs mostly associated with death (the higher the coefficient, the more correlated the drug is with patient death), and 10 drugs most associated with patient discharge alive (the lower the coefficient, the more correlated the drug is with patient discharge)

Drug administered	Coefficient
Vasoactive drugs	0.12
Sedatives	0.05
Erythropoietin	0.04
Furosemide	0.03
Amiodarone	0.03
Bromopride	0.03
Scopolamine	0.03
Blood components	0.02
Antipyretics	0.02
IV corticosteroids	0.02
Tramadol	− 0.03
Beta− blocker anti hypertensive	− 0.03
Risperidone	− 0.03
Budesonide/formoterol	− 0.04
Antivirals	− 0.04
Oral corticosteroids	− 0.06
Anticoagulants	− 0.06
Angiotensin receptor blockers (ARB)	− 0.07
Angiotensin-converting enzyme inhibitors (ACEi)	− 0.08
Ferrous sulphate	− 0.09

Table 2 shows the result of the feature selection process considering patients of all ages, regardless of the date of onset of symptoms. These are the 20 most important out of 209 drugs administered. The higher the number, the more correlated the drug is with patient death, and the lower the score, the more correlated the drug is with the patient discharge. The dataset with the complete results is available at Additional file 1: Table S1. The twenty drugs mostly correlated with outcome are available at Additional file 1: Table S2. Both supplementary tables are included in Additional file 1.doc.

## Discussion

By evaluating drug prescriptions in severe hospitalized COVID-19 patients it was possible to find correlations between the drugs used and patient outcomes (death or discharged alive). Anticoagulants were used very frequently during all phases of the disease, but were associated with good prognosis only when used after the first week of symptoms. Other drugs such as antibiotics were very frequently prescribed, especially in the early phase of the disease, but were not correlated with outcome, corroborating data that bacterial co-infections are infrequent [16], thus there have been an overuse of antibiotics as initial treatment for COVID-19 with no additional benefit.

There are different ways of looking at these results. Drugs can be indicators of the patients’ clinical condition. For example, patients who used sedative drugs were probably patients who are on mechanical ventilation or under palliative care. Furosemide may be associated with patients who have renal dysfunction. Thus, our results showing vasoactive drugs as predictors of death are logical and reinforce the adequacy of our method and results. Computer models such as the ones used to learn from the data can only infer from what they are exposed to. In this case, there is a strong correlation between some drugs and the outcome of death, but not necessarily a causal relation. Correlations may help physicians to have insights about treatments and drugs to be tested in clinical trials, but studies such as ours cannot alone support causality between treatments and the outcome. On the other hand, a model such as this can help physicians and hospital managers in the analysis of patient prognosis based on simple and objective information.

These results also indicate a means to seek for drugs that can potentially affect the clinical course of COVID-19. There is an important risk of thromboembolic phenomena associated with COVID-19. Autopsy findings in COVID-19 patients often show microthrombosis in the microvasculature [17].The adequate management of anticoagulants may be associated with a better outcome in patients with COVID-19 [3, 4].

Dexamethasone, a corticosteroid, has been shown to decrease mortality in hospitalized patients with COVID-19 who required respiratory support [18]. In our study, corticosteroid therapy showed no correlation with outcome. This may be explained by the absence of robust studies of corticosteroid therapy in the first months of the pandemic. In this situation, corticosteroids were probably used late, and for the most severe patients. Later on in the pandemic, with greater evidence in the literature, corticosteroid prescription changed.

We decided to evaluate the drugs during different periods starting at the onset of symptoms due to the relatively unique physiopathology of COVID-19. In the initial weeks of the disease, manifestations are mainly due to the activity of the virus. However, after 7 days of disease, the predominance of symptoms refers to inflammatory mechanisms [19]. Thus, it is important to evaluate drugs in these scenarios, as different drugs may be useful in different phases. In our study, almost all drugs prescribed during the first week of symptoms were poorly correlated with outcome. This is expected because in this period probably drugs that had a direct antiviral activity or antibodies may have some impact on outcome [20, 21]. On the other hand, drugs started in the second week and directed towards treating complications of COVID-19, such as anticoagulants, showed a significant correlation with survival.

Interesting to note that antibiotics were widely prescribed during the four time periods evaluated, however their correlation with prognosis was almost non-existent. This is probably explained because co-infection with COVID-19 is rare, estimated to be 3.5%, thus initial antibiotic treatment is probably futile. Conversely, a meta-analysis reported that 14.3% of COVID-19 patients developed secondary infection that is more probable only after the first week, when severe cases require the use of invasive devices and invasive procedures. Even so, antibiotic use was not correlated with outcome, suggesting that bacterial superinfection is not determinant to outcome and may be a marker of severity and prognosis [16]. Despite this, an increase in antimicrobial consumption during the COVID-19 pandemic was described [22, 23].

Finally, we felt that it was adequate to divide the patients in age groups. The prognosis of COVID-19 varies based on the age of the infected person. Since the first reports, advanced age was associated with increased mortality. Data from Brazil described an increase of more than three times in mortality in patients over 70 years of age [24]. However, for the majority of the drugs the correlations were similar across groups. In the fourth week, the use of sedatives was correlated with death in the oldest group. which may be a marker of palliative care, or may be indicative of worse prognosis of old patients using mechanical ventilation in the late phase of COVID-19.

An important limitation of our study is that it was observational. The study included all patients treated in the hospital for whom there was enough information about the start of their symptoms and drugs administered. Therefore, causal inferences are limited and may cause bias. There are important differences between the inferences that computational models can make and the direct correlation between the administration of some drug and the outcome. In summary, the correlation analysis uses only data from drug prescriptions, and outcome. These do not cover all clinical data environments, but this is the first important step for understanding the complex correlation analysis between patients with COVID-19 and their electronic health records.

## Conclusions

The advance in the processing power of computers in the last years has allowed the algorithms of Artificial Intelligence to emerge as a viable approach to analyze huge amounts of data. However, traditional techniques such as Neural Networks, Support Vector Machine and Random Forest usually require the domain knowledge in order to allow adequate feature selection extraction. A more recent approach is using techniques called deep learning that allows learning without an explicit step of features extraction. Meantime, this approach usually requires a bigger amount of varied instants in the training phase. Independently of the approach used, the correct recording as well as the use of preprocessing techniques are necessary to understand and prepare data for processing, and these tasks are possible only if multidisciplinary teams work together. Although several challenges are still present, data analysis certainly is experiencing an advance never seen before with the application of Artificial Intelligence algorithms.

In conclusion, we achieved an important result in the area of Artificial Intelligence, as we were able to establish a correlation between concrete variables in a real and extremely complex environment of clinical data from COVID-19. Our results are an initial and promising contribution in decision-making and real-time environments to support resource management and forecasting prognosis of patients with COVID-19, although decisions on treatment should be backed predominantly by randomized controlled trials. Furthermore, analyses such as these may point to promising strategies for controlled trials. Our next steps include the correlation analysis using daily medical and nurse evaluations, demographics, vital signs, laboratory tests, and evaluations of radiologic films in order to provide a software framework to support clinical predictive analysis of patients with COVID-19.

## Supplementary Information

**Additional file 1 Table S1** List of drugs prescribed, frequency of prescription, and proportion of patients discharged alive who used the drug and** Table S2** Twenty drugs mostly correlated with outcome according to feature selection --- age groups and weeks since the onset of symptoms.

## Data Availability

The datasets analyzed during the current study are not publicly available, since they were extracted from Hospital das Clínicas patients’ electronic health records. Data on patients are protected by medical confidentiality. The ethics approval includes the assurance that patient data will be analyzed in aggregate and that individual patient data will not be released. Data requests can be addressed to the corresponding author, who will evaluate the possibility of fulfilling the request considering the patients’ privacy.

## Abbreviations

CI: Central Institute
COVID-19: Coronavirus disease 2019
HC: Hospital das Clínicas
ICU: Intensive care unit
IV: Intravenous
