# Supplementary material for: Correlating drug prescriptions with prognosis in severe COVID-19: first step towards resource management
Source: BMC Med Inform Decis Mak. 2022 Sep 21;22:246. doi: 10.1186/s12911-022-01983-7 (PMC9490728; doi:10.1186/s12911-022-01983-7)
Supplement: Supplementary file 1 — Additional file 1 Table S1 List of drugs prescribed, frequency of prescription, and proportion of patients discharged alive who used the drug and Table S2 Twenty drugs mostly correlated with outcome according to feature selection --- age groups and weeks since the onset of symptoms. [file 12911_2022_1983_MOESM1_ESM.pdf]

Supplementary material — Correlating drug prescriptions with prognosis in severe COVID-19: first step towards resource management

Supplementary Table 1: List of drugs prescribed, frequency of prescription, and proportion of patients discharged alive who used the drug.

**Drug prescribed**	**Frequency**	**Proportion discharged alive (%)**
anticoagulante	3445	68.27
antibiotico	3300	66.39
antitermico	3010	64.92
omeprazol	2556	61.03
sedativo uti	2002	43.21
eletrolitos	1958	52.55
furosemida	1916	49.63
insulina	1700	55.94
corticoide iv	1652	50.79
droga vasoativa	1572	39.31
anti-psicotico	1458	55.01
bisacodil	1360	54.71
bloqueador neuro-muscular	1278	36.46
corticoide vo	1215	76.63
antiviral	1160	70.0
ondansetrona	849	73.85
metoclopramida	819	63.49
anticonvulsivante	751	64.85
hidroclorotiazida	739	58.05
estatina	728	73.08
acido acetilsalicilico	660	69.39
anti-hipertensivo bloqueador de canal de cálcio	660	80.45
metadona	629	44.83
hemocomponente	622	44.05
anti-hipertensivo ieca	519	85.36
anti-hipertensivo bra	508	90.35
vitamina	505	67.13
atropina	503	48.31
salbutamol	488	56.15
escopolamina	447	61.07
amiodarona	431	31.32
dimeticona	431	73.55
anti-hipertensivo beta-bloqueador	399	76.19
levotiroxina	392	66.33
anti-hipertensivo vasodilatador	352	66.48
sulfato ferroso	338	93.79
tramadol	333	79.58
acido folico	324	84.88
anti-hipertensivo alfa e beta-bloqueador	324	82.41
ivermectina	312	76.6
antifungico	271	47.6
espironolactona	247	72.47
formoterol+budesonida	247	84.21
bromoprida	240	55.0

Supplementary Table 1 continued from previous page

Drug prescribed	Frequency	Proportion discharged alive (%)
risperidona	235	60.85
domperidona	226	66.81
glicerofosfato	217	44.24
clopidogrel	206	67.96
zolpidem	184	90.22
sertralina	176	85.23
eritropoietina	174	64.37
ainh	162	81.48
isossorbida	147	75.51
codeina	145	85.52
ipratropio	142	71.13
fenoterol	136	65.44
metformina	128	94.53
complexo b	125	81.6
loratadina	125	79.2
imunossupressor tx	124	74.19
amitriptilina	119	74.79
tansulosina	117	82.91
fitomenadiona (vitamina k1)	111	41.44
alopurinol	98	67.35
difenidramina	97	63.92
morfina	94	60.64
dimenidrinato	92	85.87
acetilcisteina	91	76.92
sacarato de hidroxido ferrico	91	72.53
hidroxido de aluminio	89	89.89
haloperidol	84	82.14
ciclobenzaprina	83	86.75
clonidina	83	62.65
hidroxizina	81	83.95
acido tranexamico	77	37.66
glutamina	74	78.38
levomepromazina	70	85.71
fluoxetina	66	80.3
peg	63	69.84
loperamida	57	61.4
albumina	52	19.23
metaraminol	52	46.15
terbutalina	49	38.78
hidroxido aluminio+magnesio	48	89.58
tarv	46	67.39
filgrastim	43	51.16
hidroxicloroquina	43	88.37
salmeterol	40	72.5
vitamina a + d	40	95.0
adenosina	39	46.15
deslanosideo	39	30.77
budesonida	37	78.38
naloxona	37	29.73
tiotropio	37	86.49
mirtazapina	36	80.56
gliclazida	34	0.0
trombolítico	34	29.41
olanzapina	32	93.75
sulfato de zinco	31	83.87
anti-hipertensivo alfa bloqueador	29	96.55
baclofeno	28	53.57
bromazepam	27	66.67

Supplementary Table 1 continued from previous page

Drug prescribed	Frequency	Proportion discharged alive (%)
escitalopram	27	88.89
finasterida	27	81.48
colchicina	25	92.0
biperideno	23	78.26
levodopa	23	52.17
acido ursodesoxicolico	22	68.18
beclometasona	22	81.82
digoxina	20	85.0
cabergolina	18	83.33
hidroxido de magnesio	18	77.78
donepezila	17	52.94
pentoxifilina	17	5.88
venlafaxina	17	82.35
acetato de zinco	16	93.75
clortalidona	16	93.75
efedrina	16	31.25
neostigmina	16	37.5
flumazenil	15	33.33
lipase + amilase + protease	15	80.0
protamina	15	33.33
calcitriol	14	71.43
desmopressina	14	42.86
hidroxiureia	14	64.29
imunoglobulina humana 5g endovenosa	14	35.71
imunoglobulina humana anti-rho (d)	14	0.0
levetiracetam	12	91.67
milrinona	12	16.67
pamidronato	12	25.0
fenazopiridina	11	90.91
glibenclamida	10	90.0
imunossupressor	10	90.0
ciprofibrato	9	88.89
expectorante hc	8	0.0
formoterol	7	85.71
magnésio	7	85.71
oxido de magnesio	7	71.43
colestiramina	6	66.67
dimendrinato	6	83.33
duloxetina	6	83.33
ezetimibe	6	83.33
imipramina	6	66.67
nortriptilina	6	83.33
piridostigmina	6	83.33
terlipressina	6	16.67
acetazolamida	5	0.0
acido folinico	5	80.0
acido zoledronico	5	60.0
albendazol	5	80.0
carbonato de litio	5	0.0
clorfeniramina	5	0.0
fitomenadiona	5	60.0
metotrexato	5	80.0
octreotida	5	60.0
oxibutiina	5	0.0
propafenona	5	80.0
sildenafila	5	80.0
sugamadex	5	20.0
betaistina	4	0.0

Supplementary Table 1 continued from previous page

Drug prescribed	Frequency	Proportion discharged alive (%)
cinarizina	4	0.0
glucagon	4	75.0
noretisterona	4	75.0
periciazina	4	75.0
pramipexol	4	75.0
propatilnitrato	4	0.0
tiamazol	4	50.0
tiossulfato de sodio	4	75.0
alfadornase	3	0.0
bupropiona	3	0.0
ciclofosfamida	3	0.0
flunitrazepam	3	0.0
tocilizumabe	3	0.0
tretinoina	3	66.67
acido nicotinico	2	50.0
bromocriptina	2	0.0
diosmina	2	0.0
fibrinogenio	2	0.0
flucitosina	2	50.0
hidroxipropilmetilcelulose	2	50.0
leflunomida	2	50.0
mebeverina	2	0.0
medroxiprogesterona	2	0.0
oxicodona	2	0.0
rivastigmina	2	50.0
triancinilona	2	0.0
acitretina	1	0.0
alendronato	1	0.0
azacitina	1	0.0
benznidazol	1	0.0
clomipramina	1	0.0
complexo protrombinico	1	0.0
dapsona	1	0.0
diacereina	1	0.0
doxorrubicina	1	0.0
galantamina	1	0.0
imunoglobulina de coelho antitimocitos	1	0.0
imunoglobulina humana antitetanica	1	0.0
leuprorrelina	1	0.0
metilfenidato	1	0.0
montelucaste	1	0.0
orlistate	1	0.0
pirimetamina	1	0.0
primaquina	1	0.0
riluzol	1	0.0
sulfadiazina	1	0.0
talidomida	1	0.0
teofilina	1	0.0
vincristina	1	0.0

Supplementary Table 2: Twenty drugs mostly correlated with outcome according to feature selection — age groups and intervals (weeks) since the onset of symptoms

Drug prescribed	Score	Week of symptoms	Age group
sedativo uti	0,286527894	1	0 to 50
omeprazol	0,276805119	1	0 to 50
droga vasoativa	0,271456143	1	0 to 50

Supplementary Table 2 continued from previous page

Drug prescribed	Score	Week of symptoms	Age group
bloqueador neuro-muscular	0,255178564	1	0 to 50
amitriptilina	0,204266008	1	0 to 50
fitomenadiona (vitamina k1)	0,16154288	1	0 to 50
furosemida	0,147341956	1	0 to 50
anti-psicotico	0,137122351	1	0 to 50
eletrolitos	0,1261725	1	0 to 50
risperidona	0,119617762	1	0 to 50
imunoglobulina humana anti-rho (d)	-0,050723843	1	0 to 50
corticoide vo	-0,051679233	1	0 to 50
anti-hipertensivo ieca	-0,053545712	1	0 to 50
difenidramina	-0,054064975	1	0 to 50
salbutamol	-0,055278999	1	0 to 50
anticonvulsivante	-0,056426876	1	0 to 50
metoclopramida	-0,065774523	1	0 to 50
sulfato ferroso	-0,068190643	1	0 to 50
acido folico	-0,075639533	1	0 to 50
antibiotico	-0,12552167	1	0 to 50
sedativo uti	0,175200456	1	0 to 150
droga vasoativa	0,171700467	1	0 to 150
furosemida	0,155937912	1	0 to 150
omeprazol	0,145069217	1	0 to 150
bloqueador neuro-muscular	0,133650145	1	0 to 150
eletrolitos	0,118678572	1	0 to 150
corticoide iv	0,08976936	1	0 to 150
antibiotico	0,075861401	1	0 to 150
amitriptilina	0,06754859	1	0 to 150
atropina	0,066361749	1	0 to 150
complexo b	-0,036290112	1	0 to 150
sertralina	-0,036663031	1	0 to 150
formoterol+budesonida	-0,037642204	1	0 to 150
zolpidem	-0,046983628	1	0 to 150
imunoglobulina humana anti-rho (d)	-0,047921353	1	0 to 150
sulfato ferroso	-0,049634632	1	0 to 150
anticoagulante	-0,061607432	1	0 to 150
anti-hipertensivo bra	-0,07131517	1	0 to 150
acido folico	-0,071666395	1	0 to 150
hidroclorotiazida	-0,071729041	1	0 to 150
antibiotico	0,2541514	1	50 to 60
omeprazol	0,220234468	1	50 to 60
sedativo uti	0,166122699	1	50 to 60
antifungico	0,144546601	1	50 to 60
droga vasoativa	0,131640769	1	50 to 60
bloqueador neuro-muscular	0,110459	1	50 to 60
terbutalina	0,100463657	1	50 to 60
naloxona	0,100463657	1	50 to 60
deslanosideo	0,100463657	1	50 to 60
metotrexato	0,100463657	1	50 to 60
fluoxetina	-0,070766302	1	50 to 60
tramadol	-0,072564232	1	50 to 60
anti-hipertensivo ieca	-0,078073472	1	50 to 60
acido folico	-0,081883947	1	50 to 60
anti-hipertensivo bra	-0,083590748	1	50 to 60
vitamina	-0,085726457	1	50 to 60
anticonvulsivante	-0,098226119	1	50 to 60
acido acetilsalicilico	-0,113321295	1	50 to 60
levotiroxina	-0,114208048	1	50 to 60
anticoagulante	-0,240138381	1	50 to 60
eletrolitos	0,195136498	1	60 to 70

Supplementary Table 2 continued from previous page

Drug prescribed	Score	Week of symptoms	Age group
omeprazol	0,184109174	1	60 to 70
droga vasoativa	0,161195063	1	60 to 70
amiodarona	0,120145707	1	60 to 70
atropina	0,115352226	1	60 to 70
amitriptilina	0,112303294	1	60 to 70
tramadol	0,096226453	1	60 to 70
antiviral	0,095664938	1	60 to 70
salbutamol	0,095523725	1	60 to 70
anti-hipertensivo alfa e beta-bloqueador	0,092421825	1	60 to 70
bromazepam	-0,067737488	1	60 to 70
dimeticona	-0,068180782	1	60 to 70
sulfato ferroso	-0,068313824	1	60 to 70
espironolactona	-0,071510656	1	60 to 70
eritropoietina	-0,08520969	1	60 to 70
acido folico	-0,085250927	1	60 to 70
anti-hipertensivo beta-bloqueador	-0,088206771	1	60 to 70
anti-hipertensivo bloqueador de canal de cálcio	-0,092579981	1	60 to 70
zolpidem	-0,095696155	1	60 to 70
antitermico	-0,099132894	1	60 to 70
furosemida	0,216712625	1	70 to 110
sedativo uti	0,211115466	1	70 to 110
bloqueador neuro-muscular	0,132008399	1	70 to 110
droga vasoativa	0,114480412	1	70 to 110
corticoide iv	0,106428772	1	70 to 110
eritropoietina	0,090154905	1	70 to 110
hemocomponente	0,081533975	1	70 to 110
acido tranexamico	0,072991674	1	70 to 110
protamina	0,072727737	1	70 to 110
levotiroxina	0,056709648	1	70 to 110
deslanosideo	-0,063573347	1	70 to 110
zolpidem	-0,063913491	1	70 to 110
tiotropio	-0,064211239	1	70 to 110
dimeticona	-0,064743184	1	70 to 110
sertralina	-0,0797211	1	70 to 110
formoterol+budesonida	-0,082781961	1	70 to 110
estatina	-0,097561203	1	70 to 110
anti-hipertensivo bra	-0,146818265	1	70 to 110
hidroclorotiazida	-0,181434705	1	70 to 110
anticoagulante	-0,200443479	1	70 to 110
furosemida	0,298963663	2	0 to 50
sedativo uti	0,241562865	2	0 to 50
droga vasoativa	0,241537238	2	0 to 50
hemocomponente	0,18632762	2	0 to 50
bloqueador neuro-muscular	0,185709065	2	0 to 50
fitomenadiona (vitamina k1)	0,149525836	2	0 to 50
corticoide iv	0,134658919	2	0 to 50
eletrolitos	0,133923512	2	0 to 50
alopurinol	0,121909058	2	0 to 50
sugamadex	0,120262198	2	0 to 50
anticoagulante	-0,033227763	2	0 to 50
antiviral	-0,035003831	2	0 to 50
dimeticona	-0,035593425	2	0 to 50
loratadina	-0,037435674	2	0 to 50
zolpidem	-0,040626096	2	0 to 50
dimenidrinato	-0,042512166	2	0 to 50
corticoide vo	-0,044855314	2	0 to 50
sulfato ferroso	-0,046833345	2	0 to 50
antitermico	-0,047775865	2	0 to 50

Supplementary Table 2 continued from previous page

Drug prescribed	Score	Week of symptoms	Age group
ondansetrona	-0,066708318	2	0 to 50
furosemida	0,271072362	2	0 to 150
sedativo uti	0,257101525	2	0 to 150
bloqueador neuro-muscular	0,25194672	2	0 to 150
droga vasoativa	0,246826621	2	0 to 150
eletrolitos	0,215784609	2	0 to 150
corticoide iv	0,133215457	2	0 to 150
hemocomponente	0,118102663	2	0 to 150
omeprazol	0,107807313	2	0 to 150
albumina	0,091548913	2	0 to 150
insulina	0,084874485	2	0 to 150
hidroxido aluminio+magnesio	-0,036147141	2	0 to 150
hidroxicloroquina	-0,036307984	2	0 to 150
metformina	-0,03658888	2	0 to 150
metoclopramida	-0,041131352	2	0 to 150
ciclobenzaprina	-0,041320607	2	0 to 150
hidroxido de aluminio	-0,048126753	2	0 to 150
dimenidrinato	-0,050158233	2	0 to 150
ondansetrona	-0,059571028	2	0 to 150
ivermectina	-0,065948337	2	0 to 150
anticoagulante	-0,094396155	2	0 to 150
eletrolitos	0,349318089	2	50 to 60
bloqueador neuro-muscular	0,33068553	2	50 to 60
sedativo uti	0,296931301	2	50 to 60
droga vasoativa	0,232255022	2	50 to 60
insulina	0,228379289	2	50 to 60
omeprazol	0,21793625	2	50 to 60
furosemida	0,199820942	2	50 to 60
corticoide iv	0,17639507	2	50 to 60
hemocomponente	0,121617971	2	50 to 60
glicerofosfato	0,112800925	2	50 to 60
anti-hipertensivo bloqueador de canal de cálcio	-0,051866062	2	50 to 60
anti-hipertensivo vasodilatador	-0,054279632	2	50 to 60
ondansetrona	-0,055601115	2	50 to 60
dimenidrinato	-0,05825508	2	50 to 60
acido acetilsalicilico	-0,065515156	2	50 to 60
anticoagulante	-0,075945603	2	50 to 60
anti-hipertensivo beta-bloqueador	-0,076635999	2	50 to 60
difenidramina	-0,078654613	2	50 to 60
tramadol	-0,08043754	2	50 to 60
ivermectina	-0,089273929	2	50 to 60
bloqueador neuro-muscular	0,330999645	2	60 to 70
furosemida	0,277026021	2	60 to 70
droga vasoativa	0,254926358	2	60 to 70
eletrolitos	0,205031648	2	60 to 70
corticoide iv	0,170440036	2	60 to 70
sedativo uti	0,165703428	2	60 to 70
hemocomponente	0,141321394	2	60 to 70
omeprazol	0,128239958	2	60 to 70
albumina	0,106282789	2	60 to 70
difenidramina	0,105102386	2	60 to 70
tansulosina	-0,053974605	2	60 to 70
anti-hipertensivo alfa e beta-bloqueador	-0,056502274	2	60 to 70
hidroclorotiazida	-0,059499128	2	60 to 70
anticonvulsivante	-0,06262383	2	60 to 70
ondansetrona	-0,064404003	2	60 to 70
anti-hipertensivo ieca	-0,067796864	2	60 to 70
metoclopramida	-0,069883456	2	60 to 70

Supplementary Table 2 continued from previous page

Drug prescribed	Score	Week of symptoms	Age group
domperidona	-0,070359754	2	60 to 70
clopidogrel	-0,077996092	2	60 to 70
anticoagulante	-0,123833977	2	60 to 70
sedativo uti	0,303276714	2	70 to 110
droga vasoativa	0,233364805	2	70 to 110
furosemida	0,217643025	2	70 to 110
bloqueador neuro-muscular	0,190274637	2	70 to 110
eletrolitos	0,157072806	2	70 to 110
amiodarona	0,106337918	2	70 to 110
salbutamol	0,099433457	2	70 to 110
corticoide iv	0,091651033	2	70 to 110
albumina	0,084620048	2	70 to 110
naloxona	0,084453409	2	70 to 110
hidroxido de aluminio	-0,047281456	2	70 to 110
fluoxetina	-0,047593661	2	70 to 110
acetilcisteina	-0,04766729	2	70 to 110
complexo b	-0,047944562	2	70 to 110
salmeterol	-0,058049518	2	70 to 110
ciclobenzaprina	-0,062005184	2	70 to 110
dimenidrinato	-0,062382303	2	70 to 110
sacarato de hidroxido ferrico	-0,070248778	2	70 to 110
ivermectina	-0,127680282	2	70 to 110
anticoagulante	-0,179957837	2	70 to 110
sedativo uti	0,290932521	3	0 to 50
bloqueador neuro-muscular	0,277879359	3	0 to 50
omeprazol	0,274380448	3	0 to 50
hemocomponente	0,253015001	3	0 to 50
eletrolitos	0,245115554	3	0 to 50
droga vasoativa	0,233858091	3	0 to 50
furosemida	0,211635056	3	0 to 50
corticoide iv	0,150171536	3	0 to 50
insulina	0,125060641	3	0 to 50
pentoxifilina	0,124640652	3	0 to 50
antiviral	-0,039611121	3	0 to 50
metoclopramida	-0,044189722	3	0 to 50
levomepromazina	-0,046474343	3	0 to 50
hidroxido de aluminio	-0,046595777	3	0 to 50
dimeticona	-0,052547694	3	0 to 50
escopolamina	-0,052973627	3	0 to 50
sertralina	-0,054091058	3	0 to 50
acido folico	-0,062097523	3	0 to 50
sulfato ferroso	-0,063540068	3	0 to 50
anticoagulante	-0,075202001	3	0 to 50
sedativo uti	0,345100027	3	0 to 150
bloqueador neuro-muscular	0,316971099	3	0 to 150
droga vasoativa	0,263850196	3	0 to 150
furosemida	0,249343027	3	0 to 150
eletrolitos	0,237349466	3	0 to 150
hemocomponente	0,166009751	3	0 to 150
corticoide iv	0,163433994	3	0 to 150
amiodarona	0,152463823	3	0 to 150
omeprazol	0,127443742	3	0 to 150
antitermico	0,112730919	3	0 to 150
escopolamina	-0,04071399	3	0 to 150
ondansetrona	-0,047436747	3	0 to 150
dimeticona	-0,049057253	3	0 to 150
codeina	-0,050759018	3	0 to 150
corticoide vo	-0,053637575	3	0 to 150

Supplementary Table 2 continued from previous page

Drug prescribed	Score	Week of symptoms	Age group
hidroxido de aluminio	-0,054392224	3	0 to 150
sulfato ferroso	-0,056080243	3	0 to 150
anti-hipertensivo bra	-0,057641775	3	0 to 150
sertralina	-0,069843151	3	0 to 150
anticoagulante	-0,218971741	3	0 to 150
sedativo uti	0,435576218	3	50 to 60
eletrolitos	0,3355547	3	50 to 60
droga vasoativa	0,325176333	3	50 to 60
bloqueador neuro-muscular	0,324685115	3	50 to 60
antitermico	0,227141423	3	50 to 60
corticoide iv	0,223210323	3	50 to 60
furosemida	0,217621572	3	50 to 60
insulina	0,18455582	3	50 to 60
hemocomponente	0,16969203	3	50 to 60
glicerofosfato	0,130908171	3	50 to 60
dimeticona	-0,04225192	3	50 to 60
espironolactona	-0,04225192	3	50 to 60
hidroxido aluminio+magnesio	-0,050384376	3	50 to 60
dimenidrinato	-0,050509442	3	50 to 60
metoclopramida	-0,054903412	3	50 to 60
sertralina	-0,067015058	3	50 to 60
anti-hipertensivo bra	-0,067596843	3	50 to 60
anti-hipertensivo ieca	-0,074640801	3	50 to 60
corticoide vo	-0,114035885	3	50 to 60
anticoagulante	-0,227355325	3	50 to 60
sedativo uti	0,432383936	3	60 to 70
bloqueador neuro-muscular	0,329907848	3	60 to 70
droga vasoativa	0,31870765	3	60 to 70
eletrolitos	0,292068386	3	60 to 70
furosemida	0,225381049	3	60 to 70
corticoide iv	0,207043325	3	60 to 70
hemocomponente	0,147050692	3	60 to 70
amiodarona	0,145941138	3	60 to 70
omeprazol	0,135598169	3	60 to 70
bisacodil	0,121792996	3	60 to 70
fenoterol	-0,056410237	3	60 to 70
clonidina	-0,056410237	3	60 to 70
gliclazida	-0,056656609	3	60 to 70
escopolamina	-0,05672934	3	60 to 70
zolpidem	-0,057497771	3	60 to 70
loratadina	-0,064856142	3	60 to 70
codeina	-0,065249694	3	60 to 70
anti-hipertensivo bra	-0,068404557	3	60 to 70
sertralina	-0,081497948	3	60 to 70
anticoagulante	-0,115662986	3	60 to 70
bloqueador neuro-muscular	0,299954594	3	70 to 110
furosemida	0,287854291	3	70 to 110
sedativo uti	0,27909869	3	70 to 110
droga vasoativa	0,23445391	3	70 to 110
amiodarona	0,155768077	3	70 to 110
hemocomponente	0,154946398	3	70 to 110
eletrolitos	0,133191941	3	70 to 110
corticoide iv	0,128682287	3	70 to 110
antibiotico	0,107640326	3	70 to 110
antitermico	0,09339859	3	70 to 110
acido acetilsalicilico	-0,061557248	3	70 to 110
hidroxido de aluminio	-0,064989249	3	70 to 110
ciprofibrato	-0,064989249	3	70 to 110

Supplementary Table 2 continued from previous page

Drug prescribed	Score	Week of symptoms	Age group
tansulosina	-0,067570854	3	70 to 110
ivermectina	-0,072028074	3	70 to 110
espironolactona	-0,07458312	3	70 to 110
anti-hipertensivo bra	-0,084168765	3	70 to 110
estatina	-0,101316539	3	70 to 110
anti-hipertensivo bloqueador de canal de cálcio	-0,117733215	3	70 to 110
anticoagulante	-0,347692031	3	70 to 110
eletrolitos	0,342735032	4	0 to 50
sedativo uti	0,335196672	4	0 to 50
droga vasoativa	0,323957426	4	0 to 50
corticoide iv	0,293458703	4	0 to 50
bloqueador neuro-muscular	0,282490468	4	0 to 50
metadona	0,259558969	4	0 to 50
omeprazol	0,236434905	4	0 to 50
furosemida	0,194143465	4	0 to 50
hemocomponente	0,162375517	4	0 to 50
difenidramina	0,154692863	4	0 to 50
hidroxizina	-0,057430559	4	0 to 50
sulfato de zinco	-0,057563601	4	0 to 50
ciclobenzaprina	-0,063901672	4	0 to 50
vitamina	-0,064757613	4	0 to 50
antibiotico	-0,065569528	4	0 to 50
sulfato ferroso	-0,070218969	4	0 to 50
zolpidem	-0,085352738	4	0 to 50
anticonvulsivante	-0,088090512	4	0 to 50
anticoagulante	-0,096660286	4	0 to 50
corticoide vo	-0,112766581	4	0 to 50
eletrolitos	0,321262052	4	0 to 150
bloqueador neuro-muscular	0,313161431	4	0 to 150
droga vasoativa	0,312422726	4	0 to 150
sedativo uti	0,2874752	4	0 to 150
omeprazol	0,239591242	4	0 to 150
furosemida	0,234744803	4	0 to 150
corticoide iv	0,205677897	4	0 to 150
hemocomponente	0,18579915	4	0 to 150
insulina	0,158042526	4	0 to 150
amiodarona	0,132870871	4	0 to 150
sertralina	-0,054761811	4	0 to 150
hidroxizina	-0,056813186	4	0 to 150
tramadol	-0,05848687	4	0 to 150
sulfato ferroso	-0,063184064	4	0 to 150
corticoide vo	-0,064305855	4	0 to 150
hidroxido de aluminio	-0,067517726	4	0 to 150
anticonvulsivante	-0,068995474	4	0 to 150
anti-hipertensivo bra	-0,079199097	4	0 to 150
zolpidem	-0,101355433	4	0 to 150
anticoagulante	-0,201570944	4	0 to 150
bloqueador neuro-muscular	0,447876637	4	50 to 60
droga vasoativa	0,433646964	4	50 to 60
hemocomponente	0,31904573	4	50 to 60
eletrolitos	0,298494784	4	50 to 60
antibiotico	0,230259404	4	50 to 60
amiodarona	0,199484354	4	50 to 60
furosemida	0,193207964	4	50 to 60
anti-psicotico	0,181034401	4	50 to 60
insulina	0,166317511	4	50 to 60
atropina	0,166315077	4	50 to 60
anti-hipertensivo bra	-0,060522753	4	50 to 60

Supplementary Table 2 continued from previous page

Drug prescribed	Score	Week of symptoms	Age group
estatina	-0,06068008	4	50 to 60
dimeticona	-0,060751881	4	50 to 60
ivermectina	-0,062429149	4	50 to 60
sacarato de hidroxido ferrico	-0,069094128	4	50 to 60
hidroxido de aluminio	-0,078629955	4	50 to 60
metformina	-0,081516623	4	50 to 60
zolpidem	-0,08987119	4	50 to 60
tramadol	-0,091032748	4	50 to 60
anticoagulante	-0,148963506	4	50 to 60
eletrolitos	0,387459796	4	60 to 70
bloqueador neuro-muscular	0,366334212	4	60 to 70
omeprazol	0,330900303	4	60 to 70
droga vasoativa	0,271479525	4	60 to 70
furosemida	0,241315657	4	60 to 70
corticoide iv	0,209083349	4	60 to 70
hemocomponente	0,187948957	4	60 to 70
insulina	0,150922081	4	60 to 70
antitermico	0,140705294	4	60 to 70
glicerofosfato	0,132445006	4	60 to 70
codeina	-0,067636649	4	60 to 70
hidroxido de aluminio	-0,075742328	4	60 to 70
amitriptilina	-0,076595494	4	60 to 70
corticoide vo	-0,077696451	4	60 to 70
sertralina	-0,077705273	4	60 to 70
escopolamina	-0,092848707	4	60 to 70
anti-hipertensivo bra	-0,096109187	4	60 to 70
tramadol	-0,114650378	4	60 to 70
zolpidem	-0,115632869	4	60 to 70
anticoagulante	-0,271695871	4	60 to 70
sedativo uti	0,594225655	4	70 to 110
droga vasoativa	0,291134654	4	70 to 110
furosemida	0,264000336	4	70 to 110
omeprazol	0,258383731	4	70 to 110
eletrolitos	0,23812814	4	70 to 110
corticoide iv	0,22130632	4	70 to 110
bloqueador neuro-muscular	0,216535998	4	70 to 110
insulina	0,150687068	4	70 to 110
hemocomponente	0,146577217	4	70 to 110
antitermico	0,129213993	4	70 to 110
espironolactona	-0,06879244	4	70 to 110
anti-hipertensivo alfa e beta-bloqueador	-0,073861306	4	70 to 110
corticoide vo	-0,081964566	4	70 to 110
metformina	-0,082037185	4	70 to 110
tansulosina	-0,084607223	4	70 to 110
zolpidem	-0,095175922	4	70 to 110
ivermectina	-0,095987169	4	70 to 110
estatina	-0,132852493	4	70 to 110
anti-hipertensivo bra	-0,137785267	4	70 to 110
anticoagulante	-0,285345054	4	70 to 110
furosemida	0,319689616	all weeks	0 to 150
sedativo uti	0,293000891	all weeks	0 to 150
bloqueador neuro-muscular	0,283575746	all weeks	0 to 150
eletrolitos	0,224338292	all weeks	0 to 150
corticoide iv	0,198240259	all weeks	0 to 150
droga vasoativa	0,181710551	all weeks	0 to 150
amiodarona	0,152031836	all weeks	0 to 150
hemocomponente	0,145462487	all weeks	0 to 150
omeprazol	0,132579493	all weeks	0 to 150

Supplementary Table 2 continued from previous page

Drug prescribed	Score	Week of symptoms	Age group
anti-psicotico	0,129309845	all weeks	0 to 150
cabergolina	-0,035717099	all weeks	0 to 150
metformina	-0,037816969	all weeks	0 to 150
ondansetrona	-0,037995413	all weeks	0 to 150
hidroxizina	-0,03857003	all weeks	0 to 150
hidroxido aluminio+magnesio	-0,040001253	all weeks	0 to 150
imunoglobulina humana anti-rho (d)	-0,042250015	all weeks	0 to 150
ivermectina	-0,046706539	all weeks	0 to 150
dimenidrinato	-0,04727198	all weeks	0 to 150
zolpidem	-0,051560888	all weeks	0 to 150
hidroxido de aluminio	-0,063728064	all weeks	0 to 150
